# Second-line chemotherapy after gemcitabine plus nab-paclitaxel in metastatic pancreatic cancer: comparative outcomes and AI-guided treatment selection

**DOI:** 10.1093/oncolo/oyag085

**Published:** 2026-03-16

**Authors:** Letizia Procaccio, Guido Giordano, Federico Nichetti, Michele Milella, Andrea Pretta, Donatella Iacono, Monica Niger, Simona Casalino, Caterina Vivaldi, Ferdinando De Vita, Valeria Pusceddu, Matteo Landriscina, Giacomo Di Paolo, Sara Sperotto, Mariachiara Masucci, Giampaolo Tortora, Roberto Bianco, Enrico Vasile, Alberto Zaniboni, Chiara Carmen Miceli, Elena Ongaro, Elisa Giommoni, Gabriele Tinè, Giulia Barsotti, Silvia Marchesi, Mariacristina Di Marco, Silvia Ortolani, Giuseppe Aprile, Mario Scartozzi, Francesca Bergamo, Davide Melisi, Sara Lonardi

**Affiliations:** Medical Oncology 1, Veneto Institute of Oncology IOV-IRCCS, Padova, 35128, Italy; Department of Medical and Surgical Sciences, Policlinico Riuniti di Foggia, Unit of Medical Oncology and Biomolecular Therapy and University of Foggia, Foggia, 71122, Italy; Medical Oncology 1, Veneto Institute of Oncology IOV-IRCCS, Padova, 35128, Italy; Division of Oncology, Verona University and Hospital Trust (AOUI Verona), Verona, 37126, Italy; Section of Innovation Biomedicine-Oncology Area, Department of Engineering for Innovation Medicine (DIMI), University of Verona, Verona, 37134, Italy; Medical Oncology, University Hospital and University of Cagliari, Cagliari, 09124, Italy; Department of Oncology, Academic Hospital of Udine ASUFC, Udine, 33100, Italy; Medical Oncology Department, Fondazione IRCCS Istituto Nazionale dei Tumori, Milan, 20133, Italy; Investigational Cancer Therapeutics Clinical Unit, Azienda Ospedaliera Universitaria Integrata Verona, Verona, 37126, Italy; Department of Translational Research and New Technologies in Medicine and Surgery, University of Pisa, Pisa, 56126, Italy; Unit of Medical Oncology 2, Pisa University Hospital, Pisa, 56124, Italy; Division of Medical Oncology, Department of Precision Medicine, University of Campania “L. Vanvitelli”, Naples, 81100, Italy; Medical Oncology, University Hospital and University of Cagliari, Cagliari, 09124, Italy; Department of Medical and Surgical Sciences, Policlinico Riuniti di Foggia, Unit of Medical Oncology and Biomolecular Therapy and University of Foggia, Foggia, 71122, Italy; Medical Oncology 1, Veneto Institute of Oncology IOV-IRCCS, Padova, 35128, Italy; Medical Oncology 1, Veneto Institute of Oncology IOV-IRCCS, Padova, 35128, Italy; Department of Medical and Surgical Sciences, Policlinico Riuniti di Foggia, Unit of Medical Oncology and Biomolecular Therapy and University of Foggia, Foggia, 71122, Italy; Medical Oncology, Comprehensive Cancer Center, Fondazione Policlinico Universitario Agostino Gemelli, IRCCS, Rome, 00136, Italy; Medical Oncology, Università Cattolica del Sacro Cuore, Rome, 00168, Italy; Medical Oncology, University of Federico II, Naples, 80138, Italy; Unit of Medical Oncology 2, Pisa University Hospital, Pisa, 56124, Italy; Oncology, Fondazione Poliambulanza, Brescia, 25124, Italy; Division of Medical Oncology, Department of Precision Medicine, University of Campania “L. Vanvitelli”, Naples, 81100, Italy; Department of Medical Oncology, Centro di Riferimento Oncologico (CRO) IRCCS Aviano, Aviano, 33081, Italy; Medical Oncology Division, Azienda Ospedaliero-Universitaria Careggi, Firenze, 50134, Italy; Biostatistics for Clinical Research Unit, Epidemiology and Data Science Unit, Fondazione IRCCS Istituto Nazionale dei Tumori di Milano, Milan, 20133, Italy; Medical Oncology 1, Veneto Institute of Oncology IOV-IRCCS, Padova, 35128, Italy; Medical Oncology Department, Fondazione IRCCS Istituto Nazionale dei Tumori, Milan, 20133, Italy; Department of Medical and Surgical Sciences, Alma Mater Studiorum University of Bologna, Bologna, 40126, Italy; Medical Oncology Unit, IRCSS, Azienda Ospedaliero Universitaria di Bologna, Bologna, 40138, Italy; Ospedale P. Pederzoli, Casa di cura, Peschiera del Garda, 37019, Italy; Department of Oncology, Academic Hospital of Udine ASUFC, Udine, 33100, Italy; Medical Oncology, University Hospital and University of Cagliari, Cagliari, 09124, Italy; Medical Oncology 1, Veneto Institute of Oncology IOV-IRCCS, Padova, 35128, Italy; Division of Oncology, Verona University and Hospital Trust (AOUI Verona), Verona, 37126, Italy; Investigational Cancer Therapeutics Clinical Unit, Azienda Ospedaliera Universitaria Integrata Verona, Verona, 37126, Italy; Digestive Molecular and Clinical Oncology Research Unit, University of Verona, Verona, 37129, Italy; Medical Oncology 1, Veneto Institute of Oncology IOV-IRCCS, Padova, 35128, Italy

**Keywords:** pancreatic cancer, second-line chemotherapy, interpretable artificial intelligence, Nal-IRI + 5FU/LV, FOLFIRINOX, FOLFOX

## Abstract

**Background:**

International guidelines recommend 5FU/LV, Nal-IRI + 5FU/LV, FOLFIRI, FOLFOX, or (m)FOLFIRINOX as second-line (2L) chemotherapy for patients with metastatic pancreatic ductal adenocarcinoma (mPDAC) after failure of gemcitabine+Nab-paclitaxel (GnP). However, a head-to-head comparison has not been performed.

**Patients and methods:**

We conducted an observational cohort study of consecutive mPDAC patients treated with 2L chemotherapy after GnP failure at 41 Italian centers. Progression-free survival (PFS) and overall survival (OS) were compared using inverse probability of treatment weighting. Interpretable artificial intelligence methods were applied to optimize treatment allocation. A counterfactual Cox model was trained on baseline characteristics to estimate 12-month PFS under each regimen, and an Optimal Policy Tree (OPT) was derived to generate treatment recommendations, validated in a test set. Net-benefit curves evaluated clinical utility.

**Results:**

Among 704 eligible patients, 56 (8.0%) received 5FU/LV, 153 (21.7%) FOLFIRI, 209 (29.7%) FOLFOX, 209 (29.7%) Nal-IRI + 5FU/LV, and 77 (10.9%) FOLFIRINOX. FOLFIRINOX was associated with the longest PFS and OS. Median PFS was comparable among doublets (3.5 months FOLFOX, 3.6 FOLFIRI, 3.3 Nal-IRI + 5FU/LV), though Nal-IRI + 5FU/LV showed a long-term benefit. The OPT recommended Nal-IRI + 5FU/LV for patients with head/body tumors, Eastern Cooperative Oncology Group performance status (PS) 0, or CA19.9 < 109 U/mL in those with PS > 0. Net-benefit analysis showed that the OPT consistently outperformed uniform treatment strategies, achieving a 2.5 percentage-point net benefit at a threshold probability of ∼9%.

**Conclusions:**

FOLFIRINOX appears the most effective option for carefully selected, fit patients eligible for 2L chemotherapy after GnP failure. Interpretable artificial intelligence-derived treatment policies may provide superior net clinical benefit compared to uniform approaches and guide individualized therapy, warranting integration with upcoming targeted strategies such as *RAS* inhibitors.

## Graphical abstract

**Figure:**
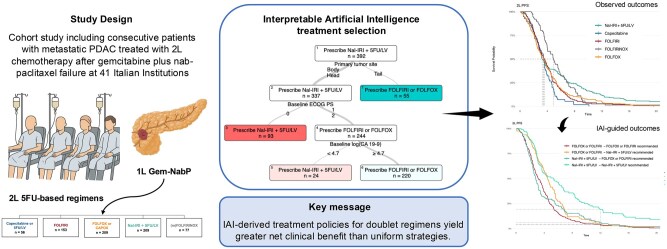

Implications for practice International guidelines recommend 5FU/LV, Nal-IRI + 5FU/LV, FOLFIRI, FOLFOX, or (m)FOLFIRINOX as second-line chemotherapy for metastatic pancreatic ductal adenocarcinoma after failure of gemcitabine+nab-paclitaxel, but head-to-head comparisons are lacking. In this multicenter cohort study, FOLFIRINOX was associated with the longest survival in highly selected patients, while doublet regimens showed similar progression-free survival, with long-term benefit only for Nal-IRI + 5FU/LV. Interpretable Artificial Intelligence (IAI) identified subgroups most likely to benefit from Nal-IRI + 5FU/LV, offering greater net clinical benefit than uniform strategies. These findings suggest FOLFIRINOX may be optimal for selected patients and highlight IAI-based models to refine treatment choices and benchmark novel *RAS* inhibitors.

## Introduction

Pancreatic ductal adenocarcinoma (PDAC) is still one of the most lethal malignancies, with increasing incidence and 5-year survival rate <15%.^1^ Its poor prognosis stems from late detection due to a lack of early markers or symptoms, high recurrence after resection and early metastatic spread leading to more than 80% of patients with unresectable disease at diagnosis. In the setting of metastatic disease, sequential systemic chemotherapy is recommended by international guidelines.^1^^,^^2^

In the last decade, FOLFIRINOX (5-fluorouracil (5FU), leucovorin (LV), irinotecan, and oxaliplatin) or modified (m)FOLFIRINOX, and gemcitabine (Gem) plus nab-paclitaxel (nP) (GnP) have represented standard first-line (1L) treatment of patients with metastatic PDAC (mPDAC), significantly improving overall survival (OS) compared to Gem monotherapy.^3^ In European countries, GnP is frequently adopted as 1L regimen due to its manageable toxicity profile and national reimbursement policies.^3–5^

Despite the limited available therapies, clinical management of patients with mPDAC has improved in the last years, enabling more patients to receive second-line (2L) therapy.^5^ After previous Gem-based treatment, the NCCN and ESMO guidelines recommend 2L fluoropyrimidine-based combinations: liposomal irinotecan (nal-IRI) plus 5FU/LV, FOLFIRI (5FU/LV, and irinotecan), (m)FOLFIRINOX or FOLFOX (5FU/LV, oxaliplatin).^2^^,^^6–8^ Nal-IRI + 5FU/LV is recommended as the best 2L option following the results of the phase 3, randomized NAPOLI-1 trial, showing its benefit in OS over 5FU/LV alone.^2^^,^^9^ The use of an oxaliplatin-based regimen yielded conflicting results in the CONKO-003 and PANCREOX studies,^10^^,^^11^ while irinotecan-based regimens are supported by non-comparative trials.^12^ Without direct comparison between these combination therapies, the choice is usually driven by multiple factors, including patient performance status (PS), comorbidities, residual toxicities from prior treatments, disease burden, 1L response, reimbursement, and ultimately patient and physician preference.^5^^,^^13^ The benefit of Nal-IRI over irinotecan or oxaliplatin in combination with 5FU/LV may not be straightforward, as suggested in indirect comparisons between 1L studies,^3^ and it has to be weighed against its clinical and financial costs. Therefore, a better selection of patients having a more favorable cost/benefit balance is needed.

Here, we present 2L survival outcomes across regimens in a large real-world Italian population of mPDAC patients treated homogeneously with 1L GnP. Based on the observed outcomes, we applied Interpretable Artificial Intelligence (IAI) methods to identify patients most likely to benefit from Nal-IRI + 5FU/LV over other doublets to maximize the benefit in this setting.

## Methods

### Study design and participants

In this observational cohort study, we included patients with mPDAC treated with 2L chemotherapy between January 1, 2013, and September 1, 2023 across 41 Italian institutions (full list in Supplementary Methods). Main inclusion criteria were: (a) age ≥ 18 years; (b) histologically/cytologically confirmed pancreatic cancer (PDAC or pancreatic carcinoma not otherwise specified, excluding neuroendocrine tumors and rare subtypes); (c) unresectable, locally advanced (per NCCN vascular criteria) or metastatic disease (collectively referred to as mPDAC); (d) prior 1L treatment with GnP, and (e) 2L treatment with 5FU-based chemotherapy. In detail, 2L regimens of interest were (a) 5FU/LV or capecitabine monotherapy, (b) FOLFOX or CAPOX, (c) FOLFIRI, (d) Nal-IRI + 5FU/LV or (e) (m)FOLFIRINOX. Patients who received at least one treatment cycle were included. To minimize confounding bias, all consecutive eligible patients were included, and no a priori sample size calculation was performed. Cases treated with Nal-IRI + 5FU/LV were included from June 2016, when a nominal use program granted access to Nal-IRI in Italy.^14^

Tumor assessment was performed every 8-12 weeks as per clinical practice, with disease status determined by local radiologists and treating physicians, based on RECIST (V.1.1) criteria.^15^ No centralized radiological review was performed. All patients were followed up until death, loss of contact, or data lock (December 1, 2023).

Demographics, baseline (before 2L treatment start) patients’ and tumor characteristics and treatment outcomes were extracted from patients charts and collected through an electronic database.

Ethics approval and written informed consent were waived, in accordance with “n.9/2016-General Authorization for Processing of Personal Data for Scientific Research Purposes-15 December 2016” (Gazzetta Ufficiale n.303, Dec 29, 2016), which permits observational studies on anonymized/pseudo anonymized data. The study was conducted in compliance with the Declaration of Helsinki and the ESMO Guidance for Reporting Oncology Real-World evidence.

Patients were not involved in the design and conduct of this research.

### Study objectives

The primary objective was to compare the efficacy of 2L treatment regimens in patients with mPDAC. The primary end point was PFS, as defined as the time between 2L chemotherapy initiation and the detection of clinical/radiological disease progression or patient death from any cause, whichever occurred first. Overall survival, as defined as the time between 2L chemotherapy initiation and patient death from any cause, was also explored. For PFS analysis, patients who were alive without disease progression were censored at their last tumor assessment. For OS analysis, patients who were alive at the end of the observation period were censored at the date of last contact.

Building on the above results and the long-term benefit (as 12-month PFS) reported in the NAPOLI-1 study subgroup of patients treated with Nal-IRI + 5FU/LV,^9^ the final aim was to develop a predictive model to identify which patients are most likely to benefit from specific chemotherapy regimens, thereby optimizing treatment efficacy while minimizing the cost of ineffective therapies.

### Statistical analyses

All statistical analyses were performed using the R software [version 4.4.2 (2024-10-31), Posit open source data science company]. Interpretable Artificial Intelligence (version 3.2.2, https://www.interpretable.ai) methods were implemented in the Julia programming language via a IAI R Interface with a dedicated license requested for the purpose of this work. A detailed list of R packages used for this manuscript is provided in the Supplementary Methods.

#### Descriptive statistics

Descriptive statistics summarized clinical and biological features by treatment group. Normality of quantitative variables was assessed using the Shapiro–Wilk test. For non-normal distributions (e.g., CA19-9), log-transformation with a pseudo-count of 1 was applied and reassessed. Variables with <10% missing data (namely primary tumor site, initial stage, number of metastatic sites, baseline Eastern Cooperative Oncology Group PS (ECOG PS), and CA19-9) were imputed using a random forest model based on other covariates (excluding outcomes), while those with >10% missingness were excluded from analysis (including resection margin status in patients who underwent surgery, and baseline lab values including neutrophil and lymphocyte counts, albumin and glycemia values).

#### Survival analysis

Median follow-up time was calculated with the reverse Kaplan–Meier (KM) method and compared among treatment groups by the Peto & Peto test. Survival curves and descriptive statistics were generated with the KM method. Group comparisons followed a two-step approach: proportional hazards were first assessed via scaled Schoenfeld residuals; as the assumption was not met (see Results), restricted mean survival time (RMST)-defined as the area under the survival curve up to time τ- was used to compare PFS and OS differences,^16^ with results reported as 95% confidence intervals (CIs). The RMST time point τ was set at 12 months from 2L treatment start, as justified above.^9^ Relative risk of progression/death was expressed as RMST ratios between treatment arms (ratio > 1 indicating improved survival). Unadjusted (univariable) and adjusted (multivariable, adopting an analysis of covariance methodology,^17^ with the following covariates: primary tumor site, prior surgery, metastatic sites, 2L baseline ECOG PS and CA19-9, and 1L PFS)^18^ models were tested. Two-tailed *P*-values < 0.05 were considered significant.

#### IPTW analysis

To address confounding in this observational study, a sensitivity analysis using Inverse Probability of Treatment Weighting (IPTW) was performed as previously described^19^: standardized differences (d values) were used to assess baseline imbalances (among the same covariates considered in multivariable models) before and after weighting, with d < 0.1 indicating negligible imbalance. A propensity score was generated via multinomial logistic regression, using the Nal-IRI + 5FU/LV arm as the reference, based on baseline covariates. These scores were used to calculate weights applied in univariable RMST models to balance treatment groups and adjust outcome comparisons. Inverse Probability of Treatment Weighting-adjusted KM curves were also generated for graphical comparison.

If significant imbalance persisted across any pairwise comparison, the treatment arm responsible (identified by d values) was excluded, and the weighting procedure repeated to ensure a balanced comparison between the remaining regimens.

#### Counterfactual predictive modelling and optimal treatment policy

Building on the observed outcomes, we next aimed to define the optimal treatment strategy among doublet regimens (Nal-IRI + 5FU/LV, FOLFIRI, and FOLFOX), to identify the correct patient for the most appropriate combination. Capecitabine and FOLFIRINOX were not considered in this specific analysis, as mono/triplet therapy were shown to be consistently inferior/superior and restricted to specific unfit/overfit populations. To achieve this, we followed a sequential two-step framework.

The dataset was first randomly split into a training set (70%) and a test set (30%). All models described below were fitted exclusively on the training set, with performance evaluated subsequently on the test set, as follows:

Step 1, counterfactual predictive modelling: to address individual variability in response to 2L regimens, we developed counterfactual predictive models, as robustly validated in prior works.^17^ These models estimate, for each patient, the outcomes probability under different treatment scenarios, simulating outcomes that cannot be directly observed in clinical practice. Given the similar early outcomes across treatment groups and the delayed benefit seen with Nal-IRI + 5FU/LV (see Results), we addressed this immortal time bias by implementing a landmark analysis at 3.5 months. We first estimated the probability of remaining progression-free at 3.5 months and then estimated the probability of remaining progression-free at 12 months conditional on surviving the first interval. This approach allowed us to incorporate early failures probabilistically and avoid bias in long-term outcome estimates. These estimates were generated using Cox proportional hazards models trained on the observational data for each treatment arm. This process yielded a counterfactual “reward matrix” containing the estimated 12-month PFS probability for every patient under each of the three doublet regimens.

Step 2: Optimal Policy Tree (OPT) training: using the reward matrix generated in Step 1, we trained an IAI model—the OPT.^20^ The OPT learned a decision policy to recommend the 2L regimen associated with the highest probability of being progression-free at 12 months based on patient characteristics. The final policy was then validated on the 30% test set to assess its generalizability, with performance evaluated by comparing predicted versus observed 12-month PFS.

We compared mean 12-month PFS under the model-recommended policy with that observed under actual treatments. We also assessed whether this personalized strategy offered greater benefit than a one-size-fits-all approach of treating all patients with Nal-IRI + 5FU/LV, using 12-month net benefit curves in both training and test sets. Full modeling framework, mathematical formulations, and implementation details are available in the Supplementary Methods. Finally, we showed the impact of OPT recommendations by plotting KM curves of real observed (not modelled) PFS of the whole cohort and of the test set only, stratifying cases by actual and OPT-recommended treatment.

## Results

### Cohort characteristics

Among 1 289 consecutive patients with mPDAC treated with 1L chemotherapy at participating centers between 2013 and 2023, 704 met eligibility criteria and were included in the study (Supplementary Figure S1A; see patient characteristics in Table 1). Among excluded cases, 182 received 1L GnP but did not receive a 2L therapy. These cases had more frequently *de novo* metastatic disease, with significantly worse ECOG PS, poorer 1L PFS and dismal OS, as expected (Supplementary Figure S1B and Supplementary Table S1).

**Figure 1.**
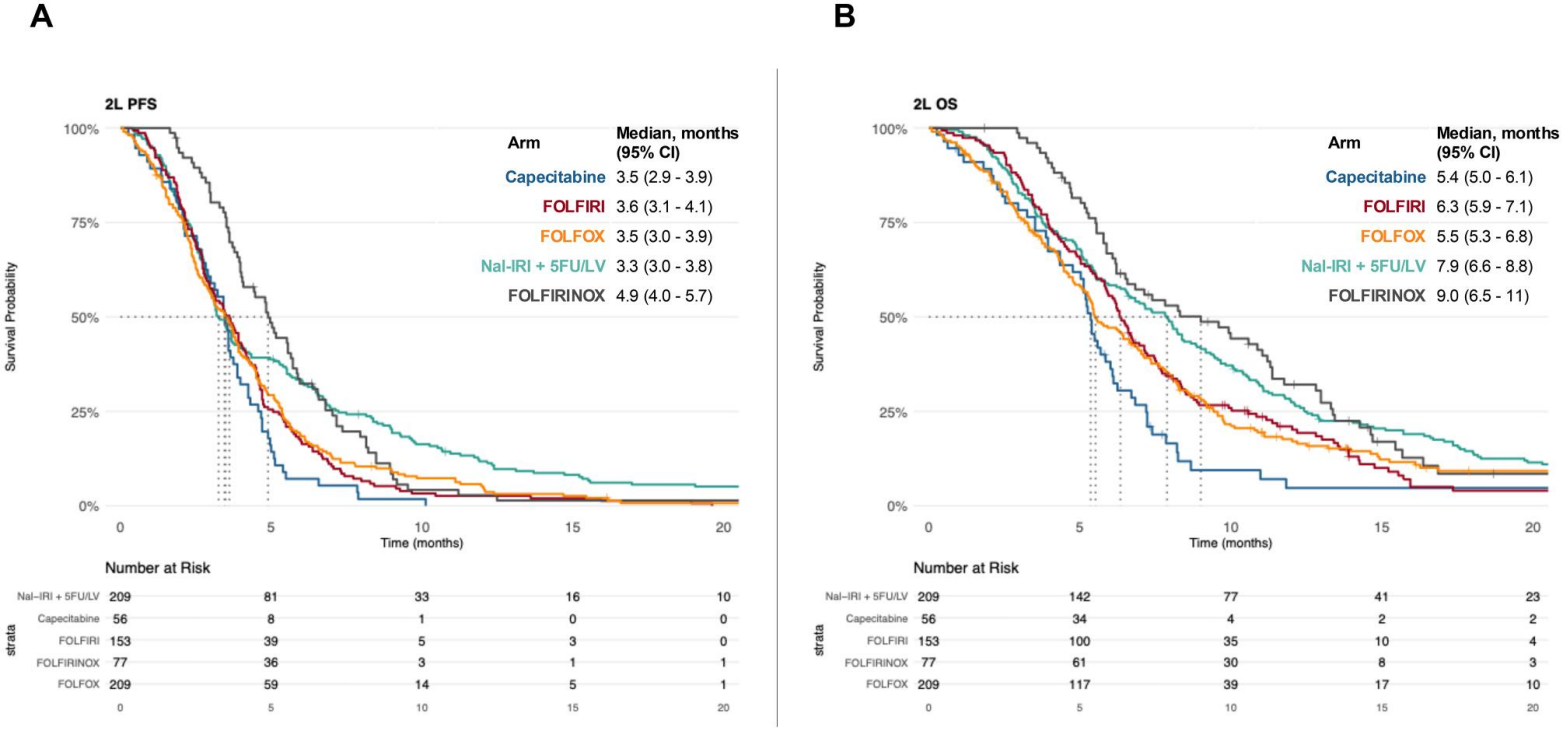
Kaplan–Meier curves for second-line chemotherapy. (A) PFS and (B) OS. Abbreviations: OS: overall survival; PFS: progression-free survival.

**Table 1.** Patients’ characteristics in the whole cohort and according to the second-line treatment regimen.

Variable	**Overall** *n* = 704	**Capecitabine** *n* = 56	**FOLFIRI** *n* = 153	**FOLFOX** *n* = 209	**Nal-IRI + 5FU/LV** *n* = 209	**FOLFIRINOX** *n* = 77	*P*-value
**Age (median—interquartile)**	67 (60-72)	72.5 (68-77)	66 (60-71)	68 (62-73)	65 (59-72)	64 (57-70)	<0.001
**Sex—female**	323 (45.9)	30 (53.6)	65 (42.5)	103 (49.3)	94 (45.0)	31 (40.3)	0.397
**Primary tumor site**							0.220
** Head**	396 (56.5)	37 (66.1)	79 (52.0)	123 (59.4)	119 (56.9)	38 (49.4)	
** Body**	201 (28.7)	12 (21.4)	53 (34.9)	59 (28.5)	52 (24.9)	25 (32.5)	
** Tail**	104 (14.8)	7 (12.5)	20 (13.2)	25 (12.1)	38 (18.2)	14 (18.2)	
** Unknown**	3		1	2			
**Initial stage**							
** IB**	4 (0.6)				3 (1.4)	1 (1.3)	
** IIA**	28 (4.0)	1 (1.8)	4 (2.6)	20 (9.7)	1 (0.5)	2 (2.6)	
** IIB**	82 (11.7)	8 (14.3)	14 (9.2)	10 (4.8)	41 (19.6)	9 (11.7)	
** III**	130 (18.5)	9 (16.1)	26 (17.0)	36 (17.4)	48 (23.0)	11 (14.3)	
** IV**	458 (65.2)	38 (67.9)	109 (71.2)	141 (68.1)	116 (55.5)	54 (70.1)	
** Unknown**	2	\	\	2	\	\	
**Prior surgery**	186 (26.4)	17 (30.4)	32 (20.9)	49 (23.4)	66 (31.6)	22 (28.6)	0.145
**Primary tumor on site or local relapse**	192 (27.3)	12 (21.4)	38 (24.8)	92 (44.0)	40 (19.1)	10 (13.0)	<0.001
**Number of metastatic sites**							0.016
** 1**	328 (47.0)	28 (50.0)	69 (45.1)	104 (50.5)	89 (43.2)	38 (49.4)	
** 2**	242 (34.7)	24 (42.9)	57 (37.3)	69 (33.5)	62 (30.1)	30 (39.0)	
** 3+**	128 (18.3)	4 (7.1)	27 (17.6)	33 (16.0)	55 (26.7)	9 (11.7)	
** Unknown**	6	\	\	3	3	0	
**Liver metastases**	493 (70.0)	40 (71.4)	103 (67.3)	146 (69.9)	147 (70.3)	57 (74.0)	0.880
**Lung metastases**	190 (27.0)	9 (16.1)	46 (30.1)	52 (24.9)	72 (34.4)	11 (14.3)	0.002
**Peritoneal metastases**	215 (30.5)	19 (33.9)	49 (32.0)	62 (29.7)	57 (27.3)	28 (36.4)	0.588
**Baseline Eastern Cooperative Oncology Group PS**							<0.001
** 0**	194 (27.6)	2 (3.6)	34 (22.2)	37 (17.7)	94 (45.0)	27 (35.1)	
** 1**	408 (58.0)	31 (56.4)	98 (64.1)	140 (67.0)	99 (47.4)	40 (51.9)	
** 2**	101 (14.4)	22 (40.0)	21 (13.7)	32 (15.3)	16 (7.7)	10 (13.0)	
** Unknown**	1	1	\	\	\	\	
**Baseline log(CA19-9)**	6.6 (4.9 - 8.4)	7.6 (6.2 - 9.1)	6.3 (5.0 - 7.9)	6.5 (5.0 - 8.1)	6.6 (4.1 - 8.9)	6.8 (5.1 - 8.2)	0.194
** Unknown**	54	13	15	12	7	7	
**First line progression-free survival** (**PFS)**							0.156
** PFS < 3**	104 (14.8)	13 (23.2)	22 (14.4)	26 (12.4)	30 (14.4)	13 (16.9)	
** PFS 3-9**	415 (58.9)	31 (55.4)	89 (58.2)	139 (66.5)	113 (54.1)	43 (55.8)	
** PFS > 9**	185 (26.3)	12 (21.4)	42 (27.5)	44 (21.1)	66 (31.6)	21 (27.3)	
**Third line chemotherapy**	219 (31.1)	15 (26.8)	78 (51.0)	81 (38.8)	1 (0.5)	44 (57.1)	<0.001
**Only patients with second-line failure (*n* = 689)**	219 (31.8)	15 (26.8)	78 (51.0)	81 (40.1)	1 (0.5)	44 (60.3)	<0.001

Of the eligible patients, 56 (8%) received capecitabine (or 5FU/LV), 153 (21.7%) FOLFIRI, 209 (29.7%) FOLFOX (or CAPOX), 209 (29.7%) Nal-IRI + 5FU/LV, and 77 (10.9%) (m)FOLFIRINOX. As expected, baseline characteristics varied across treatment groups. Patients treated with capecitabine were older, had poorer ECOG PS, and shorter 1L PFS. FOLFOX was more often used in patients with primary tumors still in place or with local relapse, while FOLFIRINOX was associated with fewer lung metastases. Patients receiving Nal-IRI + 5FU/LV had better baseline ECOG PS and more frequent lung involvement. Notably, only 1 patient (0.5%) in this group proceeded to third-line therapy. Median 1L PFS was 6.3 months (95% CI 6.0-6.7) across the cohort, with no significant differences between regimens except for a modest but statistically significant advantage of Nal-IRI + 5FU/LV over capecitabine (Supplementary Table S2, Supplementary Figure S2). Median follow-up was 52.9 months (Interquartile rangeb [IQR] 16.5-56.1), significantly longer for patients treated with Nal-IRI + 5FU/LV, likely affecting the third-line proportions observed (Table 1). Follow-up times by regimen were: 21.4 (IQR 21.4-not reported [NR]) for capecitabine, 54.3 (11.6-NR) for FOLFIRI, 26.7 (13.2-NR) for FOLFOX, 56.1 (52.9-NR) for Nal-IRI + 5FU/LV, and 18.7 (14.5-NR) for FOLFIRINOX.

**Figure 2.**
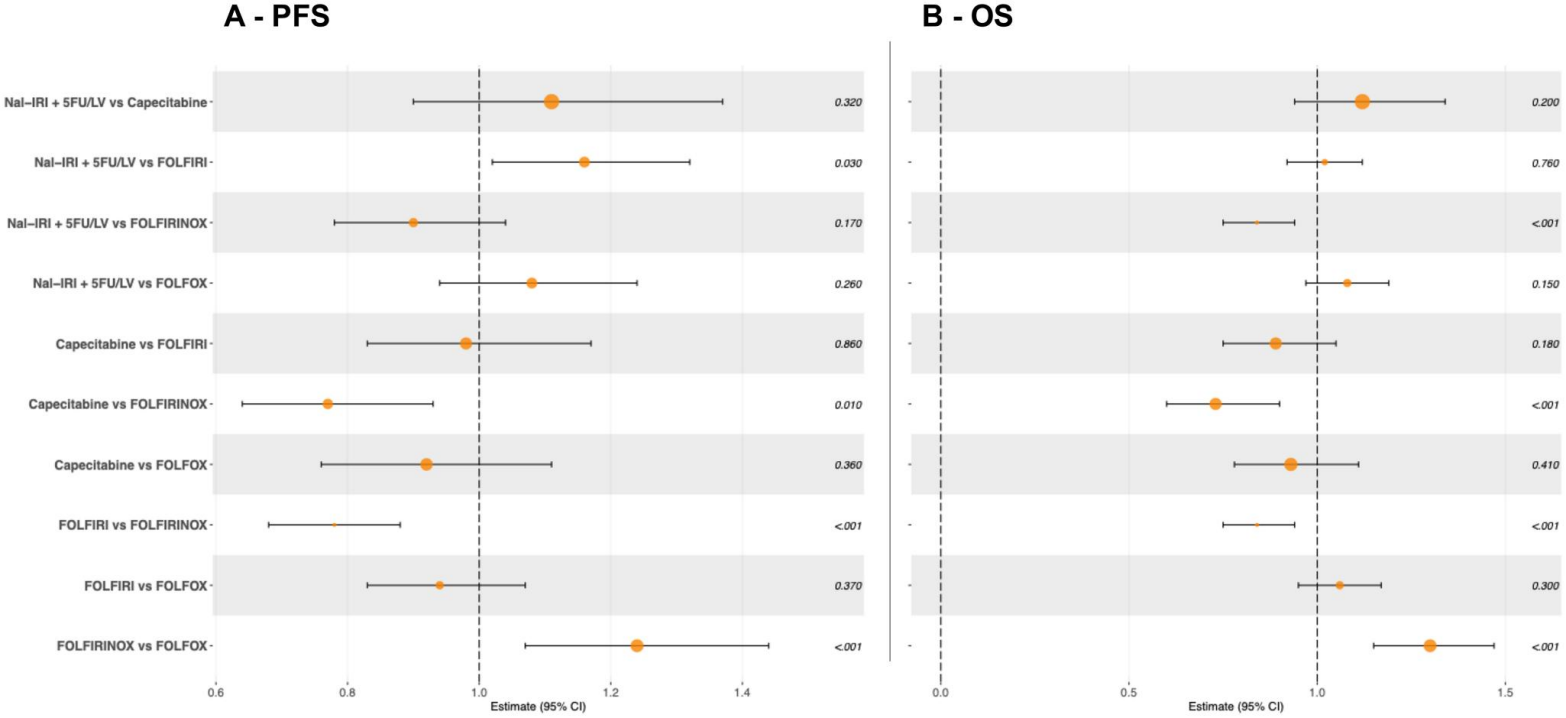
Multivariable-adjusted pairwise analysis of RMST for (A) PFS and (B) OS. Abbreviations: OS: overall survival; PFS: progression-free survival; RMST: restricted mean survival time.

### Survival outcomes

Median PFS in the overall cohort was 3.6 months (95% CI 3.4-3.8) and was similar across capecitabine, FOLFIRI, FOLFOX, and Nal-IRI + 5FU/LV, but longer in patients treated with FOLFIRINOX (Supplementary Table S3). Survival curves confirmed a significant PFS benefit with FOLFIRINOX (Figure 1A). Notably, curves for FOLFIRI, FOLFOX, and Nal-IRI + 5FU/LV overlapped up to ∼3.5 months, after which Nal-IRI + 5FU/LV diverged, suggesting a subgroup of patients deriving long-term benefit. This pattern violated the proportional hazards assumption (global chi-square *P* < .001). Restricted mean survival time was significantly longer for FOLFIRINOX and Nal-IRI + 5FU/LV in univariable analysis (Supplementary Figure S3). Adjusted RMST models confirmed FOLFIRINOX as superior to all regimens except Nal-IRI + 5FU/LV, which maintained a significant advantage over FOLFIRI (Figure 2A, Supplementary Table S4).

**Figure 3.**
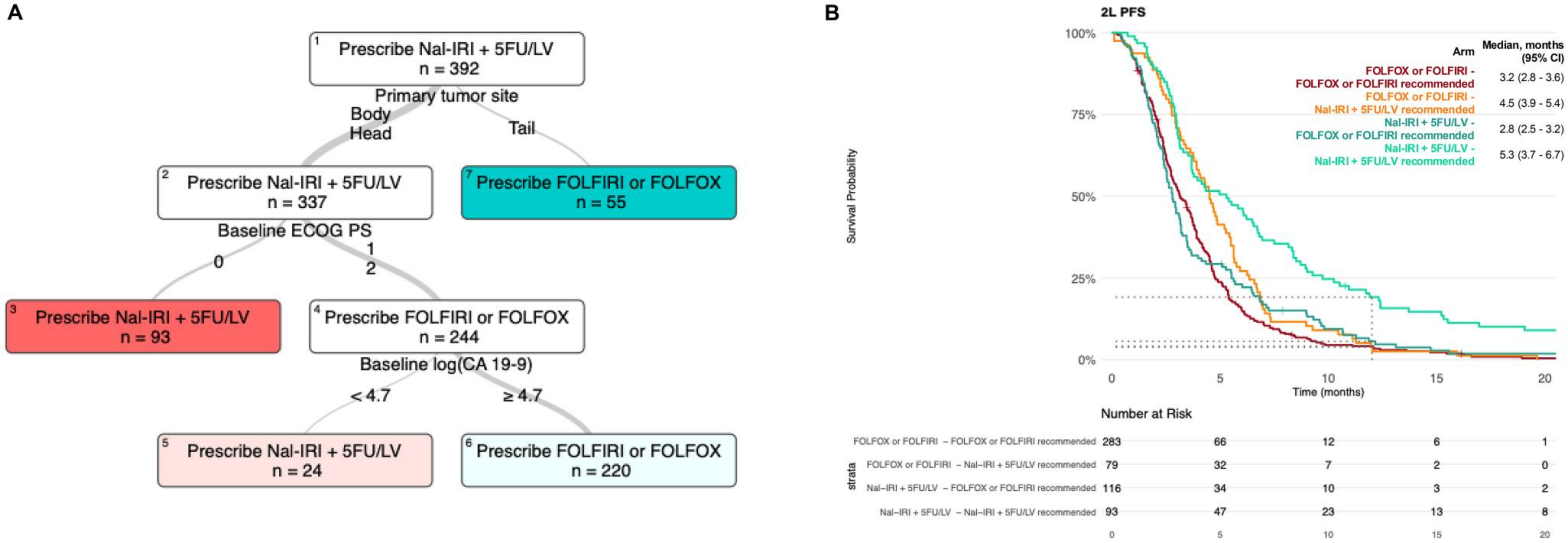
(A) 12-month PFS Optimal Policy Tree for second-line doublet chemotherapy regimens for patients with advanced PDAC. (B) Kaplan–Meier curves for second-line PFS stratified by actual versus recommended treatment per OPT (based on primary tumor site, ECOG PS, and CA19-9). Abbreviations: ECOG PS: Eastern Cooperative Oncology Group Performance Status; PDAC: pancreatic ductal adenocarcinoma. PFS: progression-free survival.

For OS, median survival was longer for patients treated with FOLFIRINOX (9.0 [95%CI 6.5-11] months) and Nal-IRI + 5FU/LV (7.9 [95% CI 6.6-8.8] months) as compared to those receiving capecitabine (5.4 [95% CI 5.0-6.1] months), FOLFIRI [6.3 (95% CI 5.9-7.1) months] and FOLFOX [5.5 (95% CI 5.3-6.8) months] (Figure 1B). As with PFS, the proportional hazards assumption was violated (*P* = .012), and RMST analysis again favored FOLFIRINOX and Nal-IRI + 5FU/LV over other regimens, while monotherapy showed the shortest OS (Supplementary Table S5). In unadjusted RMST ratio analysis, FOLFIRINOX was significantly superior to all others; Nal-IRI + 5FU/LV also showed superiority, except when compared with FOLFIRI, where the difference was not significant (Supplementary Figure S3). Adjusted analysis confirmed the OS benefit of FOLFIRINOX, while Nal-IRI + 5FU/LV showed a non-significant trend toward longer survival compared to other doublets and monotherapy (Figure 2B, Supplementary Table S5).

### IPTW analysis

Several covariates showed significant imbalance between 2L treatment arms based on standardized mean differences (Supplementary Table S6). After multi-arm propensity score weighting, imbalance persisted—particularly in the capecitabine and FOLFIRINOX groups, which showed the widest weight distributions. Inverse Probability of Treatment Weighting-adjusted KM curves for PFS revealed an early benefit with capecitabine and FOLFIRINOX compared to other regimens, which initially showed overlapping effects. Over time, the curves diverged, with a long-term benefit emerging in a subset of patients treated with Nal-IRI + 5FU/LV (Supplementary Figure S4, Supplementary Table S7). Restricted mean survival time analysis confirmed FOLFIRINOX as the most effective, with significant differences over capecitabine and FOLFIRI; OS analysis showed similar, though more modest, trends (Supplementary Table S8).

Given the imbalance, a sensitivity analysis limited to patients treated with doublets was performed, yielding comparable findings in a more balanced setting (Supplementary Table S6, Supplementary Figure S5, Supplementary Tables S9 and S10).

### Optimal treatment policy

The results above highlight the importance of identifying which patients—excluding those eligible for triplet regimens or unfit for combination therapies—are most likely to benefit from Nal-IRI + 5FU/LV over other doublets. To this aim, the IAI-based OPT (Figure 3) suggests that Nal-IRI + 5FU/LV should be administered primarily to patients with tumors localized to the head or body of the pancreas and a baseline ECOG PS of 0. Additionally, it is recommended for patients with an ECOG PS of 1-2 when log(CA19-9 + 1) is <4.7 (i.e., <109 U/mL). For all other patients, the policy tree indicates that FOLFIRI or FOLFOX is the preferred treatment, achieving comparable benefits with lower costs. These recommendations are consistent with the observed effect of immortal time bias at 3.5 months, where patients with more advanced disease show no significant benefit from Nal-IRI + 5FU/LV over other doublets.

Net benefit analysis (Supplementary Figure S6) suggests that the OPT-based policy outperforms uniform strategies of treating all patients with either Nal-IRI + 5FU/LV or FOLFIRI/FOLFOX in this modelling framework. Across both training and test sets, it consistently delivers higher net benefit across a range of threshold probabilities. For instance, to achieve a net benefit equivalent to a 12-month PFS gain of 2.5%, the probability is 4.8% under a treat-all Nal-IRI + 5FU/LV strategy and increases to 8.3% with the OPT-guided policy.

To visualize the impact of these recommendations, we stratified observed PFS and OS by actual versus OPT-recommended treatment (based on primary tumor site, ECOG PS, and CA19-9) in the whole cohort and in the test cohort only. The resulting KM curves (Figure 3B, Supplementary Figures S7 and S8) show that patients who received Nal-IRI + 5FU/LV contrary to OPT recommendations had outcomes comparable to those treated with FOLFIRI/FOLFOX, indicating a lack of added benefit. Conversely, patients who received FOLFIRI/FOLFOX despite being OPT candidates for Nal-IRI + 5FU/LV—though they surpassed the 3.5-month PFS mark, likely due to more indolent disease—showed potentially improved long-term outcomes only when actually treated with Nal-IRI + 5FU/LV.

## Discussion

Our study presents and compares 2L outcomes from the largest reported cohort of patients with mPDAC homogeneously treated with 1L GnP, to our knowledge. By limiting inclusion to patients progressing on GnP—whereas prior studies focused on smaller cohorts treated with various gemcitabine-based regimens^21–25^—we reduced selection bias and increased the reproducibility of our findings in current clinical landscape. Moreover, we deliberately excluded terminally ill patients for whom 2L treatment would not have provided any benefit.

In both unadjusted and adjusted analyses, (m)FOLFIRINOX was associated with significantly longer PFS and OS compared to other 2L options. The observed advantage is biologically consistent with the increased dose intensity of a triplet regimen. However, as expected, patients receiving this intensive regimen had more favorable baseline features, suggesting the 'biological’ benefit is inextricably linked to the fitness required to receive it. Previous retrospective series and small phase I-II trials^22^^,^^26–29^ report a median OS of 7.0-10.3 months with 2L FOLFIRINOX, aligning with our findings. However, toxicity remains a concern, and more than half of patients previously treated with GnP are ineligible for FOLFIRINOX in routine practice.^30^ Reflecting this, only 10% of our cohort received (m)FOLFIRINOX. Therefore, while encouraging, these data should be taken cautiously, suggesting that a 2L triplet association in a continuum of care strategy should be reserved to fit and young (<70 years) patients with mPDAC. At the other end of the spectrum, our data reaffirm that 5FU/LV or capecitabine monotherapy represents a suboptimal treatment, being associated with the shortest PFS and OS.

When restricting the analysis to doublets, survival curves for FOLFIRI, FOLFOX, and Nal-IRI + 5FU/LV overlapped until ∼3.5 months. Beyond this point, Nal-IRI + 5FU/LV diverged, identifying a subgroup with long-term benefit. This was in line with NAPOLI-1,^9^ which reported 25% of patients as long-term survivors (≥12 months). Similar patterns have emerged from multiple observational studies.^14^^,^^31–33^ In NAPOLI-1, long-term survival was linked to age ≤65, Karnofsky PS ≥90, neutrophil-to-lymphocyte ratio ≤5, CA19-9 < 59× the upper normal limit, and absence of liver metastases.^9^

These factors were later integrated into a nomogram to predict survival with Nal-IRI + 5FU/LV.^34^ However, its clinical utility remains limited due to small sample sizes and lack of comparative data with other active regimens.

To overcome this issue, we resorted to an IAI methodology to build a robust predictive model that: (a) incorporates all relevant covariates, (b) accounts for early and late benefit differences among doublets, and (c) generates a clinically applicable decision algorithm. The resulting decision tree suggests that Nal-IRI + 5FU/LV is most beneficial for patients with low tumor burden and good clinical status. More importantly, it helps identify patients unlikely to benefit substantially (PFS <3.5 months), supporting treatment de-escalation to avoid unnecessary clinical and financial toxicity. Although the parameters identified undoubtedly have prognostic value, the model is specifically predictive. Indeed, our models demonstrate that within the specific “better-risk” subgroup, patients treated with Nal-IRI + 5FU/LV had superior outcomes compared to those receiving FOLFOX/FOLFIRI. Consequently, the model successfully identifies the specific subgroup where the investment in a higher-cost regimen yields a tangible advantage, using clinically immediate markers that are easy for oncologists to apply in practice. Clearly, our model is a proof of concept, as we acknowledge that clinical decision-making is multifaceted. In real-world practice, choices are often limited by factors unmeasured in our model such as specific organ dysfunction, comorbidities, and insurance constraints. Moreover, as the model was specifically trained to optimize 12-month PFS, groups’ stratification at the 12-month landmark for OS is naturally less clear. Finally, while the internal validation provides a degree of robustness, these model-based findings require validation in external cohorts or prospective settings before they can be considered for clinical implementation.

Our series could provide useful insights into current practice and guide future studies. Notably, the ongoing phase 3 RASolute-302 trial (NCT06625320) is testing the panRAS inhibitor Daraxonrasib versus investigator’s choice 2L chemotherapy. The study allows various prior 1L and 2L regimens, considered equivalent in benefit. In this scenario, our study represents an important reference, on which the benefit of these trial arms and possibly our OPT will have to be confirmed.

Limitations of our study include its retrospective design and associated data gaps, including uncollected variables of potential relevance, like baseline laboratory values (e.g., renal function which may have guided treatment selection), chemotherapy dose modifications or treatment-related toxicities. Imaging assessments were conducted per clinical practice, possibly introducing variability in PFS measurement. Overall, our findings and the applicability of the OPT model are strictly limited to Italian patients who have already qualified for and received 2L chemotherapy following 1L GnP failure, thus missing a significant proportion of patients treated with different regimens^35^^,^^36^ and/or not eligible for further treatments beyond 1L.

In conclusion, our data demonstrate differential activity among 2L chemotherapy regimens after 1L GnP in mPDAC. The greatest benefit was observed in selected patients receiving FOLFIRINOX, and in a distinct subgroup treated with Nal-IRI + 5FU/LV, effectively identified using an AI-assisted model. Despite these findings, chemotherapy outcomes in mPDAC remain unsatisfactory. While awaiting novel therapies, this evidence may help refine treatment selection, optimize benefit, and reduce unnecessary cost.

## Supplementary Material

oyag085_Supplementary_Data

## Data Availability

Authors confirm that data supporting this study are available within the article and its supplementary material. Anonymized individual patient data are available upon reasonable request to the corresponding author.
