# Supplementary material for: Second-line chemotherapy after gemcitabine plus nab-paclitaxel in metastatic pancreatic cancer: comparative outcomes and AI-guided treatment selection
Source: Oncologist. 2026 Mar 16;31(4):oyag085. doi: 10.1093/oncolo/oyag085 (PMC13014169; doi:10.1093/oncolo/oyag085)
Supplement: oyag085_Supplementary_Data [file oyag085_supplementary_data.zip › 4_Supplementary Data_clean_R1.docx]

## Supplementary Data

## Supplementary Tables

**Supplementary Table 1.** Patients’ characteristics in the whole cohort and according to the 2L treatment regimen, including cases who did not receive any further treatment.

| **Variable** | **2L treatment**  n = 704 | **No 2L treatment**  n = 182 |
| --- | --- | --- |
| **Age (median - IQR)** | 67 (60 - 72) | 69 (61 - 75) |
| **Sex** - female | 323 (45.9) | 95 (52.2) |
| **Primary tumor site** |  |  |
| Head | 396 (56.5) | 109 (59.9) |
| Body | 201 (28.7) | 51 (28.0) |
| Tail | 104 (14.8) | 22 (12.1) |
| Unknown | 3 | \ |
| **Initial stage** |  |  |
| IB | 4 (0.6) | \ |
| IIA | 28 (4.0) | 15 (8.2) |
| IIB | 82 (11.7) | 4 (2.2) |
| III | 130 (18.5) | 19 (10.4) |
| IV | 458 (65.2) | 144 (79.1) |
| Unknown | 2 | \ |
| **Prior surgery** | 186 (26.4) | 29 (15.9) |
| **Primary tumor on site or local relapse** | 192 (27.3) | 154 (84.6) |
| **Liver metastases** | 493 (70.0) | 120 (65.9) |
| **Lung metastases** | 190 (27.0) | 49 (26.9) |
| **Peritoneal metastases** | 215 (30.5) | 46 (25.3) |
| **Baseline ECOG PS** |  |  |
| 0 | 194 (27.6) | \ |
| 1 | 408 (58.0) | 54 (29.7) |
| 2 | 101 (14.4) | 116 (63.7) |
| ≥3 |  | 12 (6.6) |
| Unknown | 1 | \ |
| **Baseline log(CA19-9)** | 6.6 (4.9 - 8.4) | 6.6 (6.4 - 7.0) |
| Unknown | 54 | 154* |
| **First line PFS** |  |  |
| PFS < 3 | 104 (14.8) | 64 (35.2) |
| PFS 3-9 | 415 (58.9) | 85 (46.7) |
| PFS > 9 | 185 (26.3) | 33 (18.1) |
| **3^rd^ line chemotherapy** | 219 (31.1) | \ |
| *data regarding baseline CA1.9 and number of metastatic sites were not collected for most patients not receiving 2L treatment | | |
|  |  |  |
|  |  |  |

**Supplementary Table 2.** First-line progression-free survival according to the 2L treatment regimen and p values of paired log rank tests

| **Arm** | **Median (95% CI)** |  | **Pairwise comparisons using Log-Rank test** | | | |
| --- | --- | --- | --- | --- | --- | --- |
| Nal-IRI + 5FU/LV | 6.8 (6.0, 7.8) |  | Nal-IRI + 5FU/LV | Capecitabine | FOLFIRI | FOLFIRINOX |
| Capecitabine | 5.6 (4.8, 7.9) | Capecitabine | 0.031 | - | - | - |
| FOLFIRI | 6.7 (6.2, 7.2) | FOLFIRI | 0.640 | 0.097 | - | - |
| FOLFIRINOX | 5.6 (5.0, 6.9) | FOLFIRINOX | 0.104 | 0.531 | 0.240 | - |
| FOLFOX | 6.2 (5.7, 6.7) | FOLFOX | 0.244 | 0.388 | 0.459 | 0.514 |

**Supplementary Table 3.** Median and restricted mean progression-free survival and overall survival according to the 2L treatment regimen

|  | **PFS** | | **OS** | |
| --- | --- | --- | --- | --- |
| **Arm** | **Median (95% CI)** | **RMST (95% CI)** | **Median (95% CI)** | **RMST (95% CI)** |
| Capecitabine | 3.5 (2.9 - 3.9) | 3.5 (3.0 - 4.0) | 5.4 (5.0 - 6.1) | 5.4 (4.7 - 6.2) |
| FOLFIRI | 3.6 (3.1 - 4.1) | 4.0 (3.6 - 4.4) | 6.3 (5.9 - 7.1) | 6.9 (6.3 - 7.4) |
| FOLFOX | 3.5 (3.0 - 3.9) | 4.1 (3.7 - 4.5) | 5.5 (5.3 - 6.8) | 6.3 (5.8 - 6.8) |
| Nal-IRI + 5FU/LV | 3.3 (3.0 - 3.8) | 5.0 (4.5 - 5.5) | 7.9 (6.6 - 8.8) | 7.5 (7.0 - 8.0) |
| FOLFIRINOX | 4.9 (4.0 - 5.7) | 5.3 (4.8 - 5.9) | 9.0 (6.5 - 11) | 8.5 (7.8 - 9.2) |

**Supplementary Table 4.** Unadjusted and adjusted RMST ratio analysis between 2L treatment regimens for PFS

|  | **Univariable** | | **Multivariable** | |
| --- | --- | --- | --- | --- |
| **Comparison** | **RMST ratio (95% CI)** | **p** | **RMST ratio (95% CI)** | **p** |
| Nal-IRI + 5FU/LV vs Capecitabine | 1.45 (1.21 - 1.72) | <.001 | 1.11 (0.90 - 1.37) | 0.320 |
| Nal-IRI + 5FU/LV vs FOLFIRI | 1.25 (1.09 - 1.44) | <.001 | 1.16 (1.02 - 1.32) | 0.030 |
| Nal-IRI + 5FU/LV vs FOLFIRINOX | 0.94 (0.81 - 1.08) | 0.390 | 0.90 (0.78 - 1.04) | 0.170 |
| Nal-IRI + 5FU/LV vs FOLFOX | 1.23 (1.06 - 1.41) | <.001 | 1.08 (0.94 - 1.24) | 0.260 |
| Capecitabine vs FOLFIRI | 0.87 (0.73 - 1.03) | 0.110 | 0.98 (0.83 - 1.17) | 0.860 |
| Capecitabine vs FOLFIRINOX | 0.65 (0.54 - 0.78) | <.001 | 0.77 (0.64 - 0.93) | 0.010 |
| Capecitabine vs FOLFOX | 0.85 (0.71 - 1.01) | 0.060 | 0.92 (0.76 - 1.11) | 0.360 |
| FOLFIRI vs FOLFIRINOX | 0.75 (0.65 - 0.86) | <.001 | 0.78 (0.68 - 0.88) | <.001 |
| FOLFIRI vs FOLFOX | 0.98 (0.85 - 1.12) | 0.740 | 0.94 (0.83 - 1.07) | 0.370 |
| FOLFIRINOX vs FOLFOX | 1.31 (1.13 - 1.51) | <.001 | 1.24 (1.07 - 1.44) | <.001 |

**Supplementary Table 5.** Unadjusted and adjusted RMST ratio analysis between 2L treatment regimens for OS

|  | **Univariable** | | **Multivariable** | |
| --- | --- | --- | --- | --- |
| **Comparison** | **RMST ratio (95% CI)** | **p** | **RMST ratio (95% CI)** | **p** |
| Nal-IRI + 5FU/LV vs Capecitabine | 1.38 (1.18 - 1.62) | <.001 | 1.12 (0.94 - 1.34) | 0.200 |
| Nal-IRI + 5FU/LV vs FOLFIRI | 1.09 (0.98 - 1.21) | 0.100 | 1.02 (0.92 - 1.12) | 0.760 |
| Nal-IRI + 5FU/LV vs FOLFIRINOX | 0.89 (0.79 - 0.99) | 0.030 | 0.84 (0.75 - 0.94) | <.001 |
| Nal-IRI + 5FU/LV vs FOLFOX | 1.19 (1.07 - 1.32) | <.001 | 1.08 (0.97 - 1.19) | 0.150 |
| Capecitabine vs FOLFIRI | 0.79 (0.67 - 0.93) | 0.010 | 0.89 (0.75 - 1.05) | 0.180 |
| Capecitabine vs FOLFIRINOX | 0.64 (0.54 - 0.76) | <.001 | 0.73 (0.60 - 0.90) | <.001 |
| Capecitabine vs FOLFOX | 0.86 (0.73 - 1.01) | 0.070 | 0.93 (0.78 - 1.11) | 0.410 |
| FOLFIRI vs FOLFIRINOX | 0.81 (0.72 - 0.91) | <.001 | 0.84 (0.75 - 0.94) | <.001 |
| FOLFIRI vs FOLFOX | 1.09 (0.97 - 1.22) | 0.160 | 1.06 (0.95 - 1.17) | 0.300 |
| FOLFIRINOX vs FOLFOX | 1.34 (1.19 - 1.51) | <.001 | 1.30 (1.15 - 1.47) | <.001 |

**Supplementary Table 6.** Balance measures in the unadjusted and adjusted populations

| **Variable** | **Maximum unadjusted balance statistic**  **across all pairwise comparisons** | **Maximum adjusted balance statistic**  **across all pairwise comparisons** | **Maximum adjusted balance statistic**  **across doublets pairwise comparisons** |
| --- | --- | --- | --- |
| **Age** | 1.026 (not balanced) | 0.338 (not balanced) | 0.119 (not balanced) |
| **Sex** | 0.133 (not balanced) | 0.153 (not balanced) | 0.025 |
| **Primary tumor site** |  |  |  |
| Body | 0.132 (not balanced) | 0.083 | 0.020 |
| Head | 0.167 (not balanced) | 0.103 (not balanced) | 0.039 |
| Tail | 0.062 | 0.090 | 0.025 |
| **Prior surgery** | 0.107 (not balanced) | 0.071 | 0.036 |
| **Primary tumor on site or local relapse** | 0.310 (not balanced) | 0.156 (not balanced) | 0.030 |
| **Liver metastases** | 0.067 | 0.183 (not balanced) | 0.042 |
| **Lung metastases** | 0.202 (not balanced) | 0.128 (not balanced) | 0.027 |
| **Peritoneal metastases** | 0.091 | 0.068 | 0.015 |
| **Baseline ECOG PS** |  |  |  |
| 0 | 0.414 (not balanced) | 0.194 (not balanced) | 0.003 |
| 1 | 0.196 (not balanced) | 0.192 (not balanced) | 0.005 |
| 2 | 0.316 (not balanced) | 0.013 | 0.006 |
| **Baseline log(CA19-9)** | 0.389 (not balanced) | 0.142 (not balanced) | 0.152 (not balanced) |
| **First line PFS** |  |  |  |
| PFS < 3 | 0.108 (not balanced) | 0.067 | 0.026 |
| PFS > 9 | 0.105 (not balanced) | 0.090 | 0.033 |
| PFS 3-9 | 0.124 (not balanced) | 0.150 (not balanced) | 0.046 |

**Supplementary Table 7.** IPTW-adjusted median and restricted mean progression-free survival and overall survival according to the 2L treatment regimen

|  | **PFS** | | **OS** | |
| --- | --- | --- | --- | --- |
| **Arm** | **Median (95% CI)** | **RMST (95% CI)** | **Median (95% CI)** | **RMST (95% CI)** |
| Capecitabine | 4.7 (3.4 - 5.0) | 4.4 (3.5 - 5.2) | 7.2 (5.6 - 8.2) | 7.2 (5.8 - 8.6) |
| FOLFIRI | 3.9 (3.4 - 4.5) | 4.4 (3.8 - 4.9) | 7.1 (6.2 - 8.3) | 7.5 (6.8 - 8.2) |
| FOLFOX | 4.0 (3.5 - 4.8) | 4.7 (4.1 - 5.4) | 7.0 (5.4 - 8.6) | 7.2 (6.5 - 7.9) |
| Nal-IRI + 5FU/LV | 3.3 (3.0 - 3.7) | 5.0 (4.5 - 5.5) | 7.9 (6.5 - 8.7) | 7.5 (7.0 - 8.1) |
| FOLFIRINOX | 5.0 (4.1 - 5.7) | 5.5 (4.7 - 6.2) | 9.0 (6.2 - 11.3) | 8.5 (7.5 - 9.4) |

**Supplementary Table 8.** Unadjusted RMST ratio analysis between 2L treatment regimens for PFS and OS after IPTW

|  | **PFS** | | **OS** | |
| --- | --- | --- | --- | --- |
| **Comparison** | **RMST ratio (95% CI)** | **p** | **RMST ratio (95% CI)** | **p** |
| Capecitabine vs FOLFIRI | 1.00 (0.80 - 1.23) | 0.990 | 0.96 (0.76 - 1.17) | 0.676 |
| Capecitabine vs FOLFIRINOX | 0.80 (0.63 - 0.99) | **0.042** | 0.85 (0.67 - 1.05) | 0.138 |
| Capecitabine vs FOLFOX | 0.92 (0.73 - 1.16) | 0.495 | 1.00 (0.79 - 1.23) | 0.984 |
| FOLFIRI vs FOLFIRINOX | 0.80 (0.67 - 0.96) | **0.016** | 0.89 (0.77 - 1.03) | 0.122 |
| FOLFIRI vs FOLFOX | 0.92 (0.76 - 1.12) | 0.411 | 1.04 (0.91 - 1.20) | 0.545 |
| FOLFIRINOX vs FOLFOX | 1.16 (0.94 - 1.42) | 0.159 | 1.17 (1.00 - 1.37) | **0.045** |
| Nal-IRI + 5FU/LV vs Capecitabine | 1.15 (0.95 - 1.42) | 0.164 | 1.05 (0.87 - 1.30) | 0.651 |
| Nal-IRI + 5FU/LV vs FOLFIRI | 1.15 (0.99 - 1.34) | 0.073 | 1.00 (0.89 - 1.13) | 0.978 |
| Nal-IRI + 5FU/LV vs FOLFIRINOX | 0.92 (0.78 - 1.09) | 0.310 | 0.89 (0.78 - 1.02) | 0.100 |
| Nal-IRI + 5FU/LV vs FOLFOX | 1.06 (0.89 - 1.28) | 0.525 | 1.04 (0.93 - 1.19) | 0.484 |

**Supplementary Table 9.** IPTW-adjusted median and restricted mean progression-free survival and overall survival according to the 2L treatment regimen considering only doublets regimens

|  | **PFS** | | **OS** | |
| --- | --- | --- | --- | --- |
| **Arm** | **Median (95% CI)** | **RMST (95% CI)** | **Median (95% CI)** | **RMST (95% CI)** |
| FOLFIRI | 3.9 (3.4 - 4.6) | 4.4 (3.9 - 4.9) | 7.1 (6.2 - 8.3) | 7.5 (6.8 - 8.2) |
| FOLFOX | 4.1 (3.6 - 4.8) | 4.8 (4.1 - 5.5) | 7.1 (5.4 - 8.6) | 7.2 (6.5 - 8.0) |
| Nal-IRI + 5FU/LV | 3.3 (3.0 - 3.7) | 5.0 (4.5 - 5.5) | 7.9 (6.5 - 8.7) | 7.5 (7.0 - 8.0) |

**Supplementary Table 10.** Unadjusted RMST ratio analysis between 2L treatment regimens for PFS and OS after IPTW considering only doublets regimens

|  | **PFS** | | **OS** | |
| --- | --- | --- | --- | --- |
| **Comparison** | **RMST ratio (95% CI)** | **p** | **RMST ratio (95% CI)** | **p** |
| FOLFIRI vs FOLFOX | 0.92 (0.76 - 1.11) | 0.376 | 1.04 (0.90 - 1.20) | 0.585 |
| Nal-IRI + 5FU/LV vs FOLFIRI | 1.14 (0.98 - 1.34) | 0.089 | 1.00 (0.89 - 1.12) | 0.977 |
| Nal-IRI + 5FU/LV vs FOLFOX | 1.05 (0.89 - 1.26) | 0.572 | 1.04 (0.92 - 1.19) | 0.557 |

**Supplementary Table 11.** Time-dependent area under the curve (AUCs) at 12 months and Brier scores of counterfactual Cox proportional hazard models. The reported metrics refer to Cox models developed separately for the training sets of patients treated with Nal-IRI + 5FU and with FOLFIRI/FOLFOX, and evaluated on their respective training and test sets. Both treatment-specific models (Nal-IRI + 5FU/LV and FOLFIRI/FOLFOX) showed good internal discrimination and low prediction error, with stable performance in their respective test cohorts and at cross-application across treatment arms.

Abbreviations: AUC: areas under the curve; CI: confidence interval.

| Model and cohort of application | AUC (95%CI) | Brier Score (95%CI) |
| --- | --- | --- |
| Training model (Nal-IRI + 5FU/LV) | 0.80 (0.69-0.91) | 0.10 (0.06-0.13) |
| Training model (Nal-IRI + 5FU/LV) on Test cohort (Nal-IRI + 5FU/LV) | 0.77 (0.58-0.97) | 0.07 (0.03-0.12) |
| Training model (FOLFIRI/FOLFOX) | 0.76 (0.65-0.88) | 0.04 (0.02-0.06) |
| Training model (FOLFIRI/FOLFOX) on Test cohort (FOLFIRI/FOLFOX) | 0.64 (0.37-0.90) | 0.04 (0.01-0.07) |
| Training model (Nal-IRI + 5FU/LV) on Training cohort (FOLFIRI/FOLFOX) | 0.73 (0.61-0.85) | 0.05 (0.03-0.07) |
| Training model (Nal-IRI + 5FU/LV) on Test cohort (FOLFIRI/FOLFOX) | 0.74 (0.55-0.94) | 0.05 (0.02-0.07) |
| Training model (FOLFIRI/FOLFOX) on Training cohort (Nal-IRI + 5FU/LV) | 0.72 (0.59-0.85) | 0.11 (0.06-0.16) |
| Training model (FOLFIRI/FOLFOX) on Test cohort (Nal-IRI + 5FU/LV) | 0.79 (0.63-0.95) | 0.07 (0.02-0.12) |

## Supplementary Figures

**Supplementary Figure 1A-B. A)** Study flow-chart. **B**) Overall survival, as evaluated from disease progression on first-line treatment, according to second-line regimen received. Patients whose death corresponded to the end of first-line therapy had 0.1 months added to their survival time for graphical purposes.

**Supplementary Figure 2.** Kaplan-Meier curves for first-line PFS according to second-line treatment regimen.

Abbreviations: PFS: progression-free survival.

**Supplementary Figure** **3A-B**. Unadjusted pairwise analysis of RMST for PFS and OS.

Abbreviations: OS: overall survival; PFS: progression-free survival; RMST: restricted mean survival time.

**Supplementary Figure** **4A-B**. IPTW-adjusted Kaplan-Meier curves for second-line PFS and OS.

Abbreviations: IPTW: Inverse Probability of Treatment Weighting; OS: overall survival; PFS: progression-free survival.

**Supplementary Figure** **5A-B**. IPTW-adjusted Kaplan-Meier curves for second-line PFS and OS considering only doublets regimens.

Abbreviations: IPTW: Inverse Probability of Treatment Weighting; OS: overall survival; PFS: progression-free survival.

**Supplementary Figure** **6A-B**. Net benefit curves for 12-month PFS. On the x-axis, threshold probabilities; on the y-axis, net benefit in terms of 12-month PFS. The red line represents the net benefit if all patients would be treated with FOLFIRI or FOLFOX (i.e., equal to zero since it is used as reference treatment group). The green line represents the net benefit if all patients were treated with Nal-IRI + 5FU/LV while the yellow line represent the net benefit treating patients with the new combined policy. The maximum net beneficial of treating all patients with Nal-IRI + 5FU/LV is around 5% with a probability very closely to 0%. At a threshold of **4.81%**, Nal-IRI + 5FU/LV achieves a **2.5 percentage-point** improvement in 12-month PFS relative to treat all patients with FOLFIRI or FOLFOX (i.e., treating all patients with Nal-IRI + 5FU/LV results in a mean increase of 12-month PFS for about 5/100 patients compared to treating all patients with FOLFIRI or FOLFOX). Its net benefit falls to zero at a threshold probability of around **7.5%**. The new AI-derived policy attains a **2.5 percentage-point** net benefit at a threshold probability of roughly **9%** (i.e., treating with the new AI-derived policy, results in a mean increase of 12-month PFS for about 9/100 patients compared to treating all patients with FOLFIRI or FOLFOX) and its benefit decreases to zero at about **15%**. Overall, the AI-derived policy provides a higher net benefit across all threshold probabilities than treating all patients with Nal-IRI + 5FU/LV.

**Supplementary Figure 7.** Kaplan-Meier curves for second-line OS stratified by actual versus recommended treatment per OPT (based on primary tumor site, ECOG PS, and CA19-9).

Abbreviations: ECOG PS: Eastern Cooperative Oncology Group Performance Status; OS: overall survival; PDAC: pancreatic ductal adenocarcinoma.

**Supplementary Figure 8A-B.** Kaplan-Meier curves for second-line PFS and OS stratified by actual versus recommended treatment per OPT (based on primary tumor site, ECOG PS, and CA19-9) in the test set. A) PFS and B) OS.

Abbreviations: ECOG PS: Eastern Cooperative Oncology Group Performance Status; PDAC: pancreatic ductal adenocarcinoma. PFS: progression-free survival.
